# Gratitude Can Help Women At-Risk for Depression Accept Their Depressive Symptoms, Which Leads to Improved Mental Health

**DOI:** 10.3389/fpsyg.2022.878819

**Published:** 2022-04-07

**Authors:** Joanna Tomczyk, John B. Nezlek, Izabela Krejtz

**Affiliations:** ^1^Institute of Psychology, SWPS University of Social Sciences and Humanities, Warsaw, Poland; ^2^Center for Climate Change and Social Transformation, SWPS University of Social Sciences and Humanities, Warsaw, Poland; ^3^College of William and Mary, Williamsburg, VA, United States

**Keywords:** gratitude, acceptance of illness, depression, anxiety, wellbeing, women’s health

## Abstract

**Introduction:**

Gratitude is commonly known as a positive emotion, but it can also be understood as a disposition—one’s inherent quality that includes being grateful for the positive aspects of one’s life and appreciating altruistic gifts. A growing body of research suggests that having a disposition of gratitude is positively related to wellbeing and psychological adjustment. The present study examined the extent to which acceptance of illness—a measure of adjustment to a distressing condition—mediated relationships between dispositional gratitude and wellbeing among women who had elevated levels of depressive symptoms.

**Methods:**

Participants were 131 women who, based on scores on the Center for Epidemiological Studies Depression scale, were at-risk for experiencing clinical depression. Thirty-five of these participants had been diagnosed as depressed at some point in their lives and 96 had not. Participants completed measures of dispositional gratitude, wellbeing, anxiety, and acceptance of illness.

**Results:**

Dispositional gratitude was positively correlated with wellbeing and was negatively correlated with depression and anxiety. Dispositional gratitude was also positively correlated with acceptance of illness. Mediational analyses found that acceptance of illness mediated relationships between dispositional gratitude and wellbeing, between dispositional gratitude and anxiety, and between dispositional gratitude and depression. Moreover, such mediation varied as a function of whether women had ever been diagnosed as depressed. Acceptance of illness was related more strongly to wellbeing for women who had been diagnosed as depressed at some time in their lives than it was for women who had never been diagnosed as depressed.

**Conclusion:**

Women with elevated depressive symptoms who were more grateful (compared to those who were less grateful) were more accepting of their condition, which was related to increased wellbeing and decreased feelings of depression and anxiety.

## Introduction

Depression affects over 280 million people in the world, which makes it one of the most common mental illnesses worldwide (World Health Organization, 2021). Moreover, depression is twice as common among women as it is among men (Brody et al., 2018), and women and men differ in how and why they are depressed (Yu, 2018). Given the greater prevalence of depression among women than among men (World Health Organization, 2017), and differences between men and women in the nature of depression (Kuehner, 2017), we thought it was appropriate to study the risk of developing depression among women, and the present study was designed to do this. Moreover, we studied women who had levels of symptoms that indicated they were at-risk of depression. To our knowledge, no previous research has examined the relationships we examined in a sample that was at-risk for clinical depression. The rationale for conducting this study was to fill this gap in the literature and to help explain how gratitude works for the benefit of this particular population because we believe it is important to understand this. The at-risk state deserves attention because it may occur just before the onset of major depressive disorder (see: a meta-analysis by Park and Yu, 2021). More consideration should be given to the identification of such a state and to finding factors that may be protective from the development of further symptoms. As the meta-analysis suggests, the at-risk stage of depression may present no specific symptoms and it is rare that at-risk individuals seek psychiatric help at this stage; thus, early screening is a “cornerstone of disease prevention” (Park and Yu, 2021, 9). We believe that not only screening but also identifying factors that are related to better outcomes for people in the at-risk stage may lead to a better understanding of this phenomenon.

We were interested specifically in relationships between gratitude and depression (and wellbeing more broadly defined), and in the mechanisms that might be responsible for relationships between these constructs. Research has consistently found that individuals who are more grateful are less depressed compared to individuals who are less grateful (Sin and Lyubomirsky, 2009; Wood et al., 2010; Allen, 2018; Portocarrero et al., 2020; Cregg and Cheavens, 2021; Iodice et al., 2021)—yet, how and why it happens are not well understood (Cregg and Cheavens, 2021). We aimed to contribute to the existing body of research by attempting to explain the mechanism underlying this relationship.

In addition to examining if such relationships existed among women with elevated symptoms of depression, we examined the role that acceptance of illness (Felton and Revenson, 1984; Kowalewska et al., 2020) might play in these relationships. We believed that acceptance of illness, understood as a type of adaptive coping to a distressing condition, would mediate relationships between gratitude and wellbeing. To provide a basis for examining these relationships, participants completed measures of dispositional gratitude, wellbeing, depression, anxiety, and acceptance of illness. More detailed descriptions of our expectations and methods are provided below.

### Depression in Women

As noted previously, about twice as many women as men become depressed at some time in their lives (e.g., Brody et al., 2018). This gender difference has been found to emerge in early adolescence and it exists for the full life span (Salk et al., 2017). Moreover, there are reasons to believe that depression among women is not the same as depression among men. For example, women tend to internalize their symptoms, displaying depressed mood, rumination, anhedonia, and feeling of worthlessness (Sloan and Sandt, 2006; Marcus et al., 2008), whereas men tend to externalize their symptoms, often displaying anger, substance abuse, and risk-taking (Bartels et al., 2013).

The greater incidence of depression among women compared to men has been attributed to numerous factors (Sloan and Sandt, 2006; Albert, 2015). For example, young girls, compared to young boys, report more interpersonal stressors, and they have a more negative perception of body, weight, and physical appearance, all of which are risk factors for depression (Hankin and Abramson, 2001). Throughout their lifetime women are exposed to more and more stressful life events (Kendler et al., 2002; Hammen, 2005), and they exhibit higher stress reactivity (Nolen-Hoeksema, 2001).

Women may also have a genetic vulnerability to depression (Sloan and Sandt, 2006), which may be attributed in part to fluctuations in hormone levels associated with the ovarian cycle (Kornstein et al., 2002). Moreover, there are types of depression that are unique to women such as premenstrual dysphoric disorder, peripartum and postpartum depression, and perimenopausal depression (Altemus et al., 2014).

### Gratitude and Depression

Gratitude has been studied both as an emotion and as a disposition. In the present study, gratitude is conceptualized as an inherent disposition that allows people to notice and appreciate what they have in life (Wood et al., 2010). People differ in how grateful they are on a dispositional level, and exhibiting a higher level of dispositional gratitude is associated with a number of benefits and positive outcomes (Wood et al., 2010).

The present study was motivated in part by the existing research that has found that the extent to which people feel grateful is negatively related to the extent to which they feel depressed. A recent meta-analysis found that people who were more grateful (compared to those who were less grateful) had lower levels of depressive symptoms and feelings (Iodice et al., 2021). Another meta-analysis, based on 51 intervention studies, found that gratitude alleviated symptoms of depression to the point that the authors recommended incorporating gratitude interventions in the professional treatment of depression (Sin and Lyubomirsky, 2009).

Although our primary interest was depression, we also examined relationships between gratitude and wellbeing defined more broadly. This broader focus was motivated by research on relationships between gratitude and wellbeing that has consistently found a positive relationship between feeling grateful and wellbeing (Ruini, 2017). This research includes studies of life satisfaction (McCullough et al., 2002; Wood et al., 2008), subjective happiness (Watkins et al., 2003), positive emotions (Emmons and McCullough, 2003; Froh et al., 2009), relationship satisfaction (Gordon et al., 2011), and self-esteem (Lin, 2015).

Consistent with those results, another meta-analysis, which focused specifically on dispositional gratitude, found that gratitude is positively related to wellbeing, satisfaction with life and happiness, and this relationship is stronger in clinical populations than in non-clinical populations (Portocarrero et al., 2020). The authors concluded that gratitude is an individual difference that is strongly related to wellbeing and that feeling grateful may be more beneficial for clinical populations than it is for healthy populations.

### Acceptance of Illness and Its Role in Depression

In addition to examining relationships between gratitude and wellbeing, we also examined the role that acceptance of illness might play in these relationships. Acceptance of illness refers to how people adjust to any condition that imposes limitations on their lives and that could make them feel useless and hopeless (Felton and Revenson, 1984), and depression is a condition that imposes limitations on people’s functioning. These limitations include, but are not limited to, neurocognitive deficits (Mohn and Rund, 2016), work impairment (Gilmour and Patten, 2007), interpersonal difficulties (McEvoy et al., 2013), and functional limitations (Hammer-Helmich et al., 2018). In addition, feelings of worthlessness are an integral part of depression (American Psychiatric Association, 2013). Even if most women in our sample were not diagnosed as clinically depressed, they suffered from the limitations that the elevated symptoms of depression imposed on their lives. In this sense, even though they may not (or not yet) have a diagnosis of an illness, they reported having a certain set of distressing symptoms that might require some types of changes in their lives. For these reasons, we believe that the concept of acceptance of illness is applicable to people who are depressed and to those who are at-risk of developing depression.

Although we are unaware of previous research that has examined relationships between symptoms of depression and acceptance of illness, there are reasons to believe that acceptance of illness is relevant to understanding feelings of depression. Felton and Revenson (1984) claimed that acceptance of illness is a manifestation of good psychological adjustment, which they defined as: (1) understanding one’s situation and condition, (2) accepting the changes that this condition brings about, (3) adjusting one’s perception of self in the light of this condition, and (4) learning strategies to cope with the distress that the condition causes (Beaumont, 2004). Cognitive-behavioral therapy for depression targets these elements while teaching people to behave and think more adaptively (Fenn and Byrne, 2013). This suggests that individual differences in acceptance of illness are related to feelings of depression, and such an assumption was part of the model that guided the present study.

We believe that acceptance of illness may provide a segue from gratitude to wellbeing. This assumption is based on previous research that has found that grateful people find it easier to accept what they have in life even if they recognize that they do not feel well or that they experience some distress. People with high dispositional gratitude, contrary to those with low dispositional gratitude, may be likely to see the world through a different lens, or to be more specific, employ other ways of cognitive processing of the events and situations that occur (Lambert et al., 2012).

### The Present Study: Expectations

The present study examined if dispositional gratitude is related to the mental health of women at-risk of developing depression, and if so, if acceptance of illness may be responsible for this relationship.

Participants were women with levels of depressive symptoms that indicated they were at-risk for experiencing depression. They completed questionnaires that measured their dispositional gratitude, wellbeing, anxiety, depression, and acceptance of illness. We expected to find the following relationships:

1. Dispositional gratitude would be positively related to mental health, with mental health defined in terms of depression, anxiety, and a more broadly focused measure of overall wellbeing.
2. Acceptance of illness would be positively related to wellbeing and negatively related to depression and anxiety.
3. Acceptance of illness would mediate relationships between dispositional gratitude and mental health.
4. On an exploratory basis, we examined whether the strength of the mediational role of acceptance of illness was moderated by participants’ diagnostic history, i.e., had they ever been diagnosed as depressed?

The first two hypotheses are relatively straightforward extensions of existing research. Our belief about the mediating role of acceptance of illness was based on the following logic. How people evaluate their immediate circumstances has a direct effect on mental health. To some extent, mental health can be thought of as a manifestation of how people evaluate their present circumstances and life situation (Diener et al., 1999).

People who are high in dispositional gratitude tend to appreciate what they have in life more than people low in dispositional gratitude (Wood et al., 2010), which makes it easier for them to accept their situation even when it is far from optimal. Such a tendency would be reflected in greater acceptance of illness. The mediation we propose is due to the fact that dispositional gratitude leads to acceptance, which in turn leads to mental health. We know of no other study that has examined the possibility that acceptance of illness might mediate relationships between dispositional gratitude and mental health. Demonstrating this could have important theoretical and practical implications, issues we address in the discussion.

Finally, we had no clear expectations regarding the possible moderating effect of diagnostic history. To our knowledge, there has been no research on this or related topics. We consider this issue in the discussion.

## Materials and Methods

### Participants

Participants were 131 women (*M*_age_ = 27.11, *SD* = 0.44) from Poland who reported exhibiting depressive symptoms at the time of the study and during the previous week. Of these 131, 35 reported having received a clinical diagnosis of depression at some point before the study (independent of the study). The remaining 96 women had never received a formal diagnosis of depression but met the criteria for being at-risk for depression in the pre-screening stage of the study (described below). Demographic information about the sample is presented in the “Results.”

### Procedure

Participants were recruited online *via* social media, including a Facebook page of the authors’ institution, and a website created for the purposes of the study. The description of the study stated that it was intended for people who had experienced five or more of the following symptoms in the previous 2 weeks. These symptoms were those described as criteria for depressive disorders in DSM 5 (American Psychiatric Association, 2013): (1) depressed mood, loss of interest, or pleasure in almost all activities, (2) significant unintentional weight loss/gain or decrease/increase in appetite, (3) sleep disturbance, (4) psychomotor changes (agitation or retardation), (5) tiredness fatigue, low energy, or decreased efficiency with which routine tasks are completed, (6) a sense of worthlessness or excessive, inappropriate, or delusional guilt, (7) impaired ability to think, concentrate, or make decisions, and (8) recurrent thoughts of death, suicidal ideation, or suicide attempts. The above symptoms cause distress or impairment in important areas of functioning and cannot be attributed to a medical condition, substance use, or loss of a loved one.

People who reported experiencing five or more of these symptoms in the previous 2 weeks were invited to complete the Center for Epidemiological Studies Depression Scale (CES-D; Radloff, 1977). The CES-D is a commonly used and well-validated screening tool for major depression disorder (Park and Yu, 2021). The scale consists of 20 items that concern symptoms of depression (see: Measures). Those who scored above 20 on the CES-D were qualified for the study. Twenty has been recommended as a conservative cutpoint (few false positive) for indicating risk for clinical depression (Vilagut et al., 2016). Next, individuals who were at-risk indicated if they were in therapy or were taking prescribed psychiatric medication. Individuals who answered yes to either of those questions were excluded from the study.

Individuals who met the above criteria then completed a set of questionnaires that included demographic information and the scales that are the focus of the present paper. After they finished the questionnaires, they were informed about the opportunity to schedule a 1-h complimentary session with a cognitive-behavioral therapist as compensation for their participation. As our participants were at-risk of experiencing severe depressive symptoms and were not in treatment, we advised taking the opportunity to talk to the professional we provided. After the study, 55 participants expressed interest in scheduling an online session with the therapist. Note that whatever therapy participants received occurred after they completed the measures that are the focus of this paper, so whatever therapy participants received could not have influenced their responses.

Participants provided written informed consent, and the procedure was approved by the Ethics Committee of SWPS University of Social Sciences and Humanities, Warsaw, Poland, approval no. 53/2020. The study was conducted in accordance with the Declaration of Helsinki.

### Measures

Copies of the items and scales we used, including the screening measures, and the data that were analyzed in this paper are available online at the Open Science Framework: https://osf.io/dvef3/?view_only=0833c094d6eb472e876cfa1d9b76c512. We administered Polish language versions of all scales. All had previously been validated in Polish samples. Alphas for all scales are presented in Table 1.

**Table 1:** Descriptive statistics and correlations for measures.

S. No.	Measure	*M*	*SD*	*α*	2	3	4	5
1.	Gratitude	4.16	1.34	0.83	0.43^***^	0.24^**^	−0.19^*^	−0.24^**^
2.	Wellbeing	2.04	0.67	0.66		0.42^**^	−0.54^**^	−0.56^**^
3.	Acceptance of illness	3.33	1.12	0.91			−0.35^**^	−0.39^**^
4.	Depression	40.65	8.32	0.82				0.48^**^
5.	Anxiety	3.07	0.44	0.73				

* *p* < 0.05;

** *p* < 0.01;

*** *p* < 0.001.

#### Dispositional Gratitude

To measure dispositional gratitude, we used the Gratitude Questionnaire (GQ6; McCullough et al., 2002). The scale consists of six items that participants rated from (1) *strongly disagree* to (7) *strongly agree*. For the full scale, the Cronbach’s alpha was 0.74.

Reliability analysis found that deleting the last item (“Long amounts of time can go by before I feel grateful to something or someone”) increased alpha to 0.83. Moreover, an exploratory factor analysis indicated that the last item loaded on a different factor than the other 5. Given this, this item was dropped from our analyses, and dispositional gratitude was represented as the mean response to the remaining five items. Similar procedure was used in previous studies on Polish samples (Krejtz et al., 2016; Tomczyk et al., 2021). In order to see if item deletion would affect the results, we carried out analyses using both the 6-item and the 5-item scale as predictors. The results were not affected—the tables that compare the models with both predictors are available in the OSF folder (see “Measures”).

#### Psychological Wellbeing

Psychological wellbeing was measured with five items taken from the WHO Quality of Life-BREF (2004), Diener et al. (1985), and Scheier et al. (1994). This measure was previously used in a study on the effect of gratitude on the wellbeing of breast cancer patients (Tomczyk et al., 2021). Examples of items are as: “I feel satisfied with myself” and “I look into the future with optimism.” Participants rated the items on a scale that had five points labeled (1) *strongly disagree* to (5) *strongly agree*. Psychological wellbeing was defined as the mean response to these five items; higher scores represented greater wellbeing.

#### Acceptance of Illness

We measured acceptance of illness using the Acceptance of Illness Scale (AIS; Felton and Revenson, 1984). Participants rated eight items using a scale with endpoints labeled (1) *agree* to (7) *disagree*. Sample item: “Because of my health, I do not feel like a worthy person.” The AIS score was calculated as the mean response to these eight items, and the items were scored such that higher scores represented a greater acceptance of illness. AIS has been typically used in chronically ill populations (Felton et al., 1984) but following the authors’ guidelines, it can be used to measure adjustment to any condition that causes psychological discomfort. Acceptance of illness is defined as “a lack of negative responses and emotions associated with a condition” (Kowalewska et al., 2020, p. 416).

#### Anxiety

We measured anxiety using ten items taken from the trait subscale of the State–Trait Anxiety Inventory (Spielberger, 1989). Sample items: “I feel nervous and restless” and “I worry too much over something that really does not matter.” Participants rated how often they had recently experienced symptoms of anxiety using a scale from (1) *almost never* to (4) *almost always.* Anxiety was defined as the mean response to these ten items, and higher scores indicated higher anxiety.

#### Depression

As discussed previously, we measured depression using the CES-D (Radloff, 1977). Participants rated each of the 20 items depending on how often they experienced the symptom during the past week, from (0) *rarely* to (3) *most of the time.* Scores on the CES-D are the sum of the 20 responses, and the higher the score, the stronger the symptoms of depression. Consistent with previous practice, we analyzed scale scores (sums of responses), not mean scores.

### Analytical Plan

The present study examined relationships among gratitude, wellbeing, and acceptance of illness, and it examined if acceptance of illness mediated relationships between gratitude and wellbeing, gratitude and anxiety, and gratitude and depression. First, we examined correlations among the measures. Then, we conducted a series of mediation analyses to estimate the direct and indirect effect of gratitude on the aforementioned outcomes, model 4 in the PROCESS macro for SPSS (Hayes, 2018). Following the advice of Hayes et al. (2017), we used PROCESS instead of structural equation modeling.

We estimated indirect effects using the percentile bootstrap method with 5,000 samples, and we report 95% confidence intervals for effects. Finally, on an exploratory basis, we conducted a series of moderated mediation analyses (model 8 in PROCESS). These analyses determined if the mediation of relationships between gratitude and wellbeing by acceptance of illness varied as a function of whether participants had received a formal diagnosis of clinical depression prior to the study.

## Results

### Demographic Information

Participants were qualified for participation based on their CES-D scores, which is a standard procedure for measuring risk for experiencing clinical depression (Radloff, 1977), and 27% of the women in our sample reported having received a formal diagnosis of clinical depression before the study. A 74.8% of our participants were unmarried, 15.3% were married, 8.4% were divorced, and 1.5% were widowed. A 22.1% had children. A 9.2% were unemployed, 36.6% were employed, 35.9% were students, 3.8% had their own business, 0.8% were on sick leave, and 13.7% had a different occupational situation they were asked to describe in an open-ended question (e.g., on maternity leave, studying and working, and working part time while taking care of a child).

### Correlations Between Gratitude, Acceptance of Illness, and Mental Health

Table 1 contains the descriptive statistics, scale reliabilities, and correlations for all measures. According to guidelines proposed by Shrout (1998), two scales were characterized by moderate reliability (Cronbach’s alpha from 0.61 to 0.80), and three scales had substantial reliability (Cronbach’s alpha above 0.81). There were no floor and ceiling effects as the means for all measures were sufficiently far from the endpoints of each scale.

The correlations were consistent with our predictions. Gratitude was positively correlated with wellbeing and acceptance of illness and was negatively correlated with depression and anxiety. Acceptance of illness was positively correlated with wellbeing, and it was negatively correlated with depression and anxiety.

### Acceptance of Illness as a Mediator of the Relationships Between Gratitude and Mental Health

We examined whether acceptance of illness mediated the relationships between (1) gratitude and wellbeing, (2) gratitude and anxiety, and (3) gratitude and depression. A summary of the results of the analyses is presented in Table 2. Note that *a* coefficient, representing the path from gratitude to acceptance of illness, was the same for all outcomes, *a* = 0.20 (*SE* = 0.07, *t* = 2.75, *p* < 0.01). All coefficients presented in tables and figures are unstandardized.

**Table 2:** Summary of mediation analyses: Indirect and direct effects of gratitude on outcomes mediated by acceptance of illness.

**Outcome**	**Indirect effect**	**Direct effect**
Wellbeing	Significant (*BootCI* [0.01, 0.08])	Significant (*p* < 0.001)
Anxiety	Significant (*BootCI* [−0.06, −0.01])	Not significant (*p* = 0.07)
Depression	Significant (*BootCI* [−0.99, −0.10])	Not significant (*p* = 0.17)

#### Wellbeing

**Figure 1:**
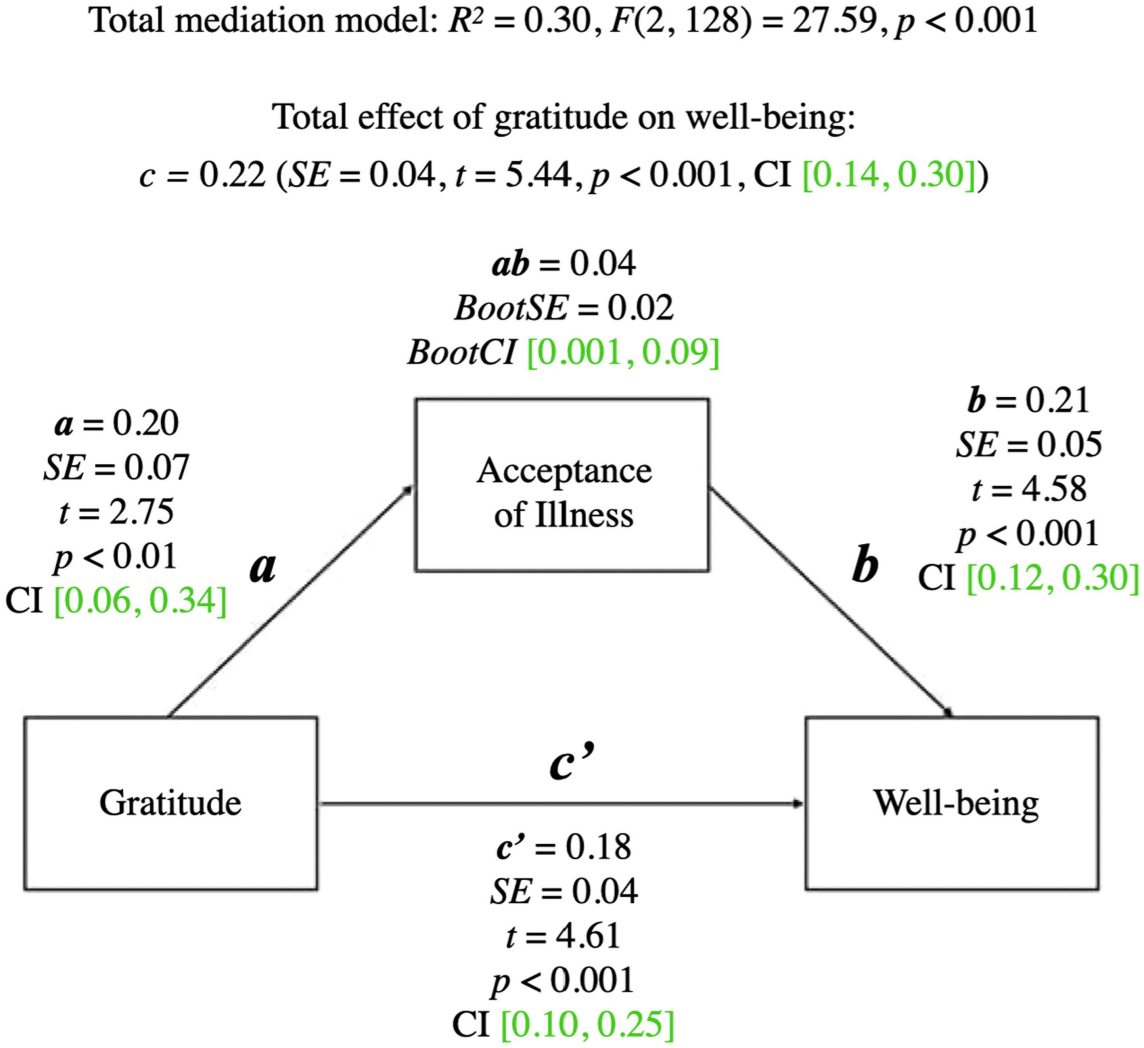
Mediation model for gratitude and wellbeing. a, effect of Gratitude on Acceptance of Illness; b, effect of Acceptance of Illness on wellbeing; ab, indirect effect of Gratitude on wellbeing; c', direct effect of Gratitude on wellbeing; c, total effect of Gratitude on wellbeing (ab + c'); CI, confidence intervals; CI, marked green indicate significance.

A summary of the mediational model for psychological wellbeing is presented below. For coefficients and additional statistics, see Figure 1 and Table 3.

**Table 3:** Model coefficients for mediation analysis with acceptance of illness as a mediator of the relationship between gratitude and mental health.

Predictor	Acceptance of illness				Wellbeing (Outcome)				Anxiety				Depression			
		Coeff.	*SE*	*t*		Coeff.	*SE*	*t*		Coeff.	*SE*	*t*		Coeff.	*SE*	*t*
Gratitude	a	0.20	0.07	2.75^**^	c ^’^	0.18	0.04	4.61^***^	c ^’^	−0.05	0.03	1.84	c ^’^	−0.07	0.52	1.38
Accept. of illness		–	–	–	b	0.21	0.05	4.58^***^	b	−0.14	0.03	4.28^***^	b	−2.43	0.63	3.86^**^
	*R^2^* = 0.06, *F*(1, 129) = 7.55, *p* < 0.01				*R^2^* = 0.30, *F*(2, 128) = 27.59, *p* < 0.001				*R^2^* = 0.17, *F*(2, 128) = 13.46, *p* < 0.001				*R^2^* = 0.14, *F*(2, 128) = 10.22, *p* < 0.001			

** *p* < 0.01,

*** *p* < 0.001.

a Relationship between gratitude and acceptance of illness.

b Relationship between acceptance of illness and mental health controlling for gratitude.

c’ Direct effect of gratitude on mental health controlling for acceptance of illness.

The total mediational model was significant (*p* < 0.001). The total effect of gratitude on wellbeing (the *c* path; *p* < 0.001) was also significant—it represented the sum of the direct effect of gratitude on wellbeing (the *c’* path; *p* < 0.001) and the indirect effect of gratitude on wellbeing through acceptance of illness (the *ab* path; *BootCI* [0.001, 0.09]). The *b* path from acceptance of illness to wellbeing was significant (*p* < 0.001). The results indicated that acceptance of illness mediated the relationship between gratitude and psychological wellbeing.

#### Anxiety

**Figure 2:**
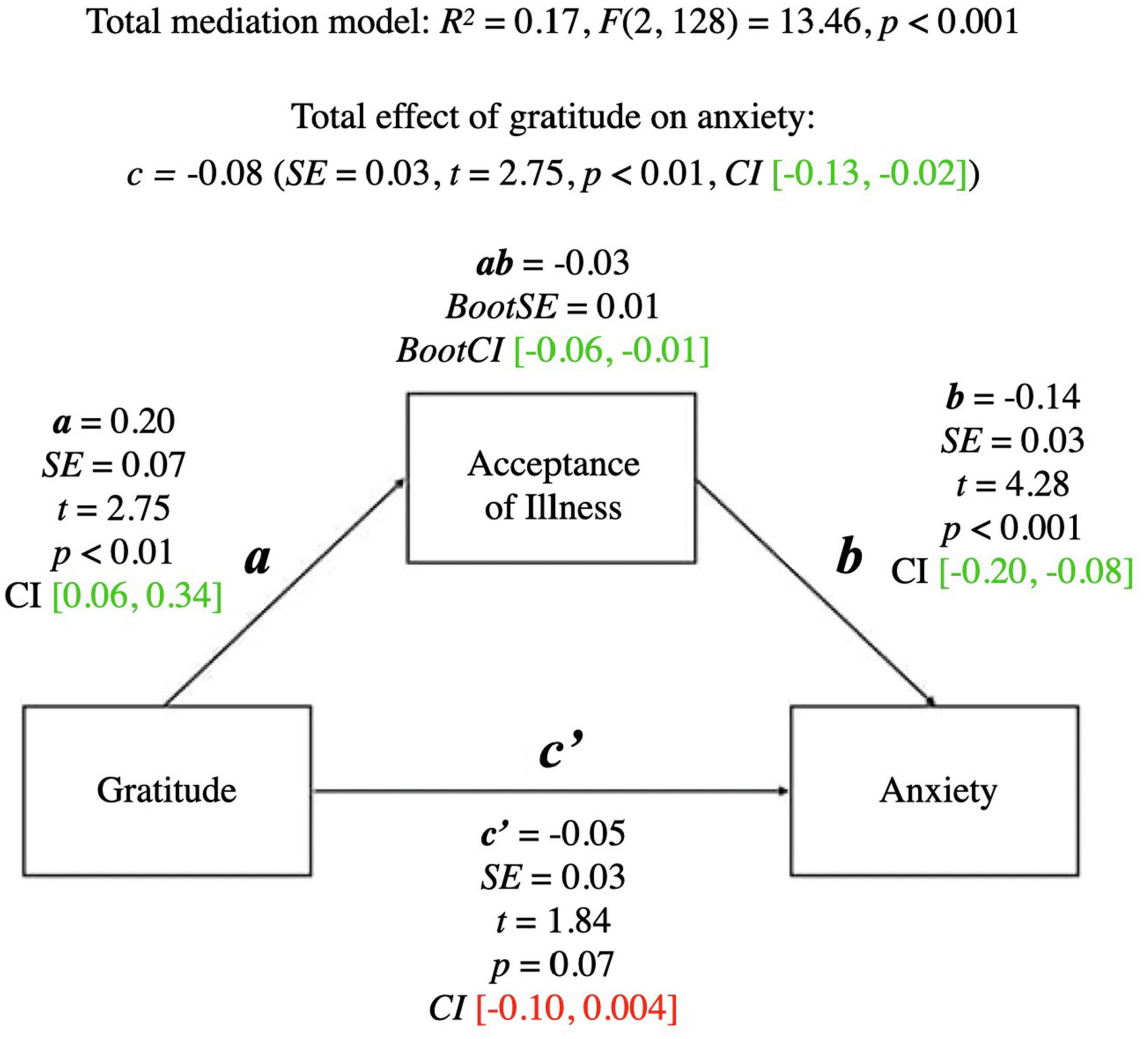
Mediation model for gratitude and anxiety. a, effect of Gratitude on Acceptance of Illness; b, effect of Acceptance of Illness on Anxiety; ab, indirect effect of Gratitude on Anxiety; c', direct effect of Gratitude on Anxiety; c, total effect of Gratitude on Anxiety (ab + c'); CI, confidence intervals; CI marked green.

A summary of the mediational model for anxiety is presented below. For coefficients and additional statistics, see Figure 2 and Table 3.

The total mediational model was significant (*p* < 0.001). The total effect of gratitude on anxiety (the *c* path; *p* < 0.01) was also significant—it represented the sum of the direct effect of gratitude on anxiety (the *c’* path; *p* = 0.07) and the indirect effect of gratitude on anxiety through acceptance of illness (the *ab* path; *BootCI* [−0.06, −0.01]). The *b* path from acceptance of illness to anxiety was significant (*p* < 0.001). The presence of the indirect effect means that acceptance of illness mediated the relationship between gratitude and anxiety.

#### Depression

**Figure 3:**
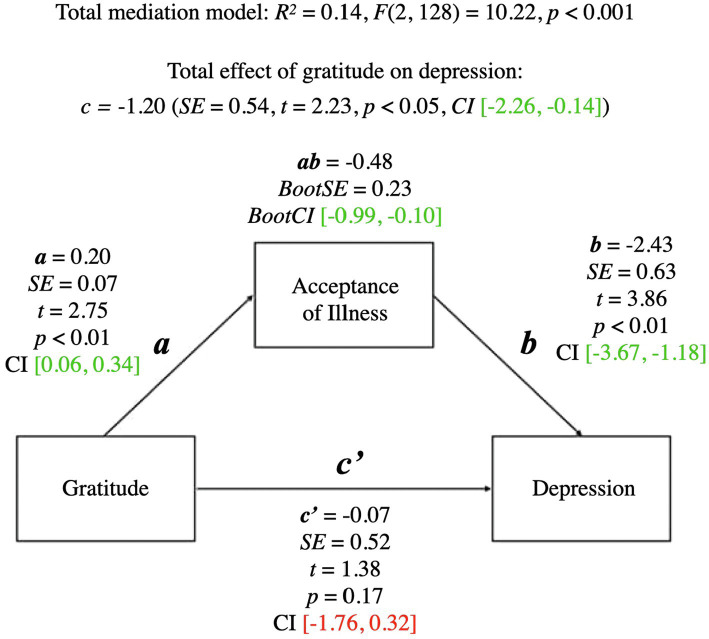
Mediation model for gratitude and depression. a, effect of Gratitude on Acceptance of Illness; b, effect of Acceptance of Illness on Depression; ab, indirect effect of Gratitude on Depression; c', direct effect of Gratitude on Depression; c, total effect of Gratitude on Depression (ab + c'); CI, confidence intervals; CI marked green indicate significance; red – lack of significance.

A summary of the mediational model for depression is presented below. For coefficients and additional statistics, see Figure 3 and Table 3.

The total mediational model was significant (*p* < 0.001). The total effect of gratitude on depression (the *c* path; *p* < 0.05) was also significant—it represented the sum of the direct effect of gratitude on depression (the *c’* path; *p* = 0.17) and the indirect effect of gratitude on depression through acceptance of illness (the *ab* path; *BootCI* [−0.99, −0.10]). The *b* path from acceptance of illness to depression was significant (*p* < 0.01). The significant indirect effect of gratitude on depression indicated that although there was no direct effect of gratitude on depression, acceptance of illness mediated the relationship between gratitude and depression.

### Diagnostic History as a Moderator of How Acceptance of Illness Mediates Relationships Between Gratitude and Mental Health

We examined if whether participants had received a diagnosis of clinical depression at some time in their lives moderated the mediation of acceptance of illness on the relationships between (1) gratitude and wellbeing, (2) gratitude and anxiety, and (3) gratitude and depression. A summary of the results of these analyses is presented in Table 4.

**Table 4:** Summary of moderated mediation analyses: Indirect and direct effects of gratitude on outcomes mediated by acceptance of illness and moderated by diagnosis.

**Outcome**	**Moderator**	**Indirect effect**	**Direct effect**
Wellbeing	No diagnosis	Not significant (*BootCI* [−0.01, 0.06])	Significant (*p* = 0.0003)
	Diagnosis	Significant (*BootCI* [0.06, 0.21])	Significant (*p* = 0.001)
Anxiety	No diagnosis	Not significant (*BootCI* [−0.04, 0.01])	Significant (*p* = 0.05)
	Diagnosis	Significant (*BootCI* [−0.14, −0.04])	Not significant (*p* = 0.98)
Depression	No diagnosis	Not significant (*BootCI* [−0.74, 0.10])	Not significant (*p* = 0.21)
	Diagnosis	Significant (*BootCI* [−2.18, −0.32])	Not significant (*p* = 0.58)

#### Wellbeing

**Figure 4:**
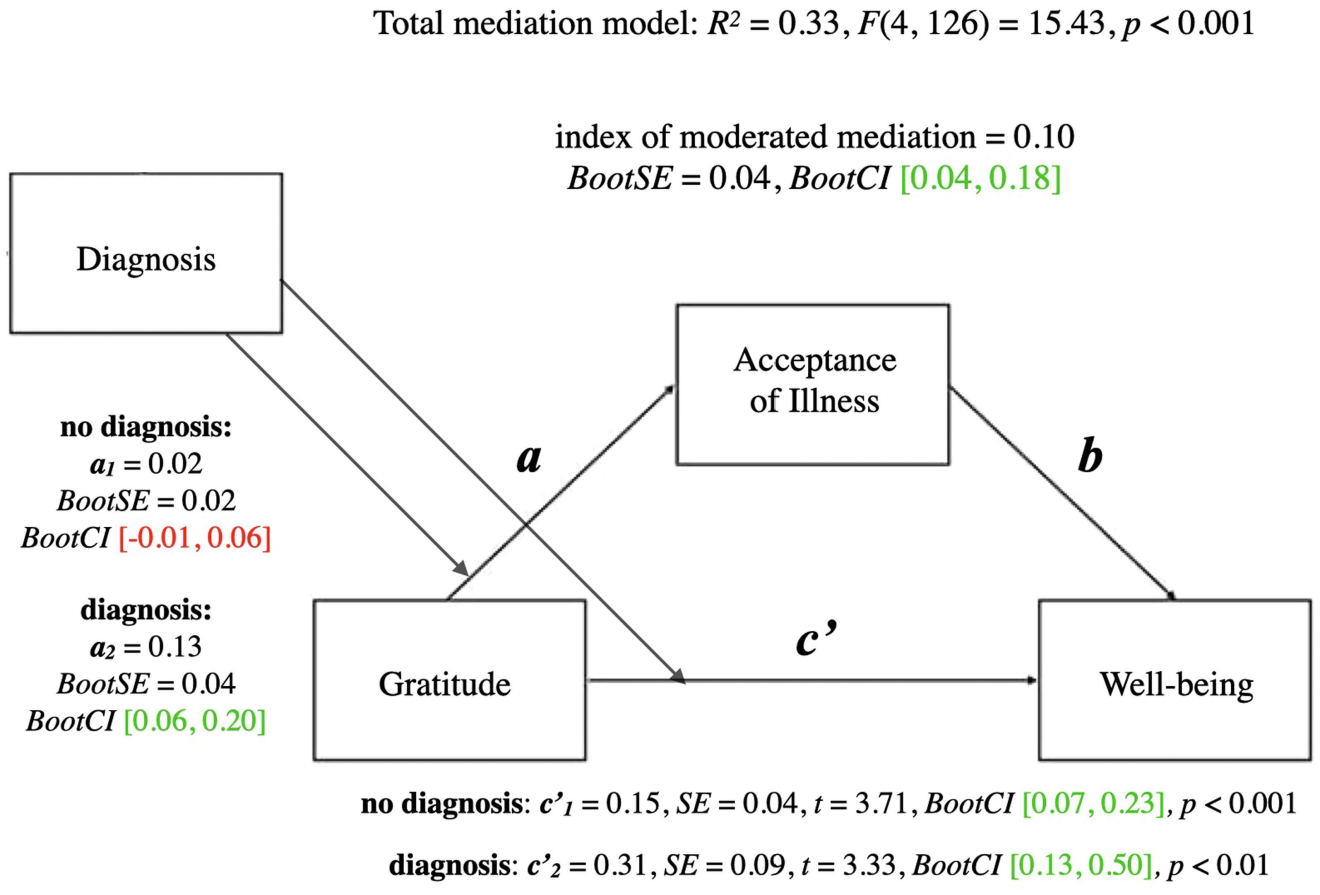
Moderated mediation model for gratitude and wellbeing with diagnosis as moderator. a, conditional indirect effect of gratitude on wellbeing through acceptance of illness; c', conditional direct effect of gratitude on wellbeing index of moderated mediation = difference between conditional indirect effects; CI marked green indicate significance; red – lack of significance.

A summary of the moderated mediation model for wellbeing is presented below. For coefficients and additional statistics, see Figure 4 and Table 5.

**Table 5:** Model coefficients for mediational analysis with acceptance of illness as a mediator of the relationship between gratitude and mental health.

		**Wellbeing (Outcome)**				**Anxiety**				**Depression**			
Moderator		Coeff.	*SE*	*CI*	*t*	Coeff.	*SE*	*CI*	*t*	Coeff.	*SE*	*CI*	*t*
No diagnosis	a	0.02	0.02	[−0.01, 0.06]	–	−0.02	0.01	[−0.04, 0.007]	–	−1.43	0.51	[−0.74, 0.10]	–
	c’	0.15	0.04	[0.07, 0.23]	3.7^***^	−0.06	0.03	[−0.12, 0.00]	1.98^*^	−0.72	0.57	[−1.85, 0.40]	1.27
Diagnosis	a	0.13	0.04	[0.06, 0.20]	–	−0.09	0.27	[−14, −0.04]	–	−0.25	0.21	[−2.46, −0.47]	–
	c’	0.31	0.09	[0.13, 0.50] ^**^	3.33	−0.002	0.07	−0.14, 0.13],	0.03	−0.74	1.32	[−3.34, 1.87]	0.56
		*R^2^* = 0.33, *F*(4, 126) = 15.43, *p* < 0.001				*R^2^* = 0.18, *F*(4, 126) = 6.87, *p* < 0.001				*R^2^* = 0.14, *F*(4, 126) = 5.05, *p* < 0.001			

*p < 0.05, **p < 0.01, ***p < 0.001.

The total mediational model for wellbeing was significant (*p* < 0.001). The conditional direct effect of gratitude on wellbeing was significant both for the people who had never been diagnosed as depressed (*p* < 0.001) and for the people who had been diagnosed at some point (*p* < 0.01). In contrast, the conditional indirect effect of gratitude on wellbeing through acceptance of illness was significant only for people who had been diagnosed with clinical depression at some point in their lives (*BootCI* [0.06, 0.20]), whereas it was not significant for people who had never been diagnosed (*BootCI* [−0.01, 0.06]). The index of moderated mediation was significant (*BootCI* [0.04, 0.18]), which indicates that the mediational relationships differed between the two groups. Acceptance of illness mediated the relationship between gratitude and wellbeing for people who had received a formal diagnosis of depression, whereas acceptance of illness did not mediate the relationship between gratitude and wellbeing for people who had not ever received a formal diagnosis of depression.

#### Anxiety

**Figure 5:**
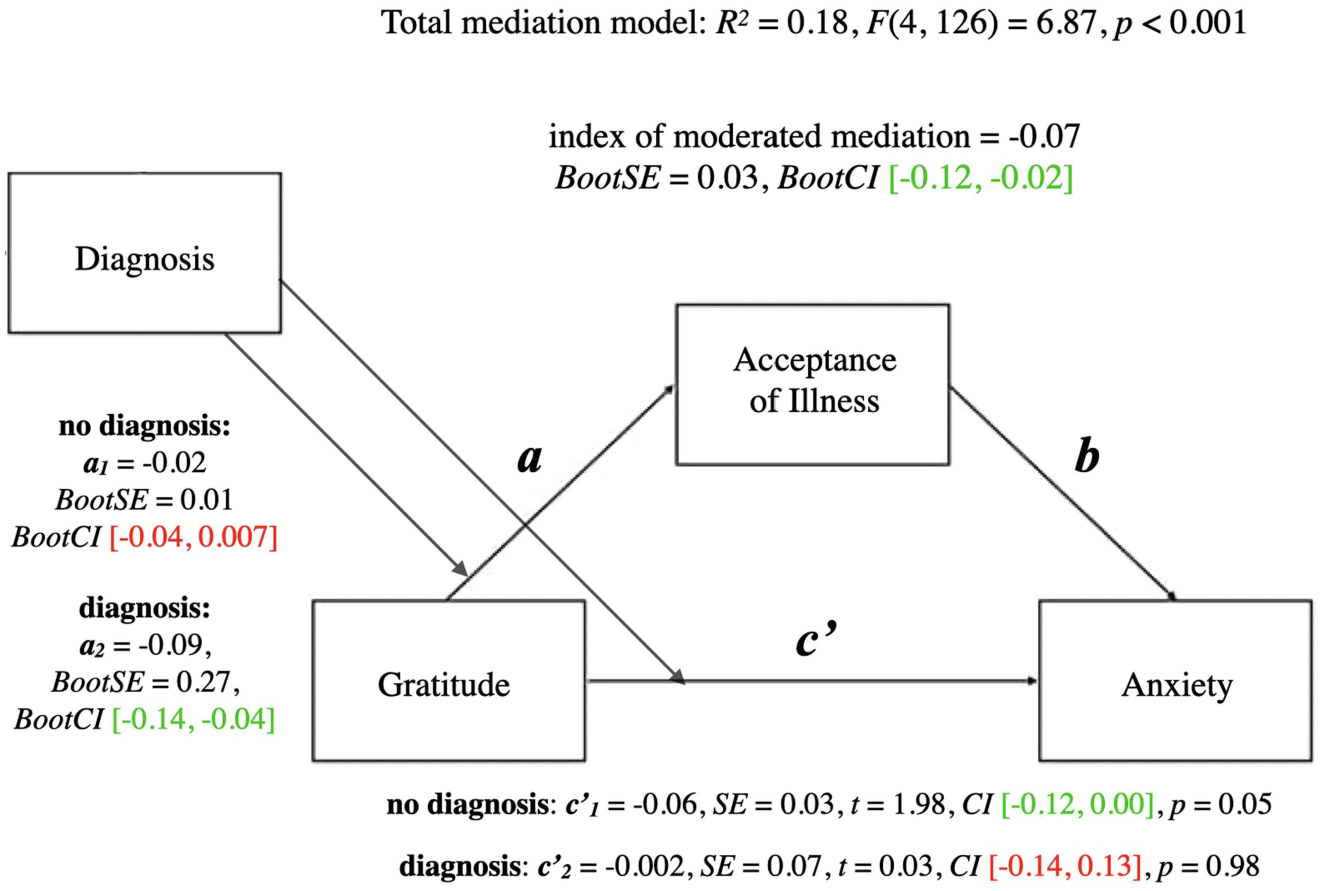
Moderated mediation model for gratitude and anxiety with diagnosis as moderator. a, conditional indirect effect of gratitude on wellbeing through acceptance of illness; c', conditional direct effect of gratitude on wellbeing index of moderated mediation = difference between conditional indirect effects CI marked green indicate significance; red – lack of significance.

A summary of the moderated mediation model for anxiety is presented below. For coefficients and additional statistics, see Figure 5 and Table 5.

A similar analysis with anxiety as an outcome found that the total mediational model was significant (*p* < 0.001). The conditional direct effect of gratitude on anxiety was verging on significant for people who had not been diagnosed as depressed as some point (*p* = 0.05), whereas it was not significant for people who had been diagnosed at some point in their lives (*p* = 0.98). In contrast, the conditional indirect effect of gratitude on anxiety through acceptance of illness was significant for people who had been diagnosed as depressed at some point depression (*BootCI* [−14, −0.04]), whereas it was not significant for people who had not been diagnosed as depresses at some point (*BootCI* [−0.04, 0.007]). Moreover, the index of moderated mediation was significant (*BootCI* [−0.12, −0.02]), indicating that the mediational relationships differed between the two groups. Similar to the results of the analyses of wellbeing, acceptance of illness mediated the relationship between gratitude and anxiety for people who had received a formal diagnosis of depression, whereas acceptance of illness did not mediate the relationship between gratitude and anxiety for people who had not ever received a formal diagnosis of depression.

#### Depression

**Figure 6:**
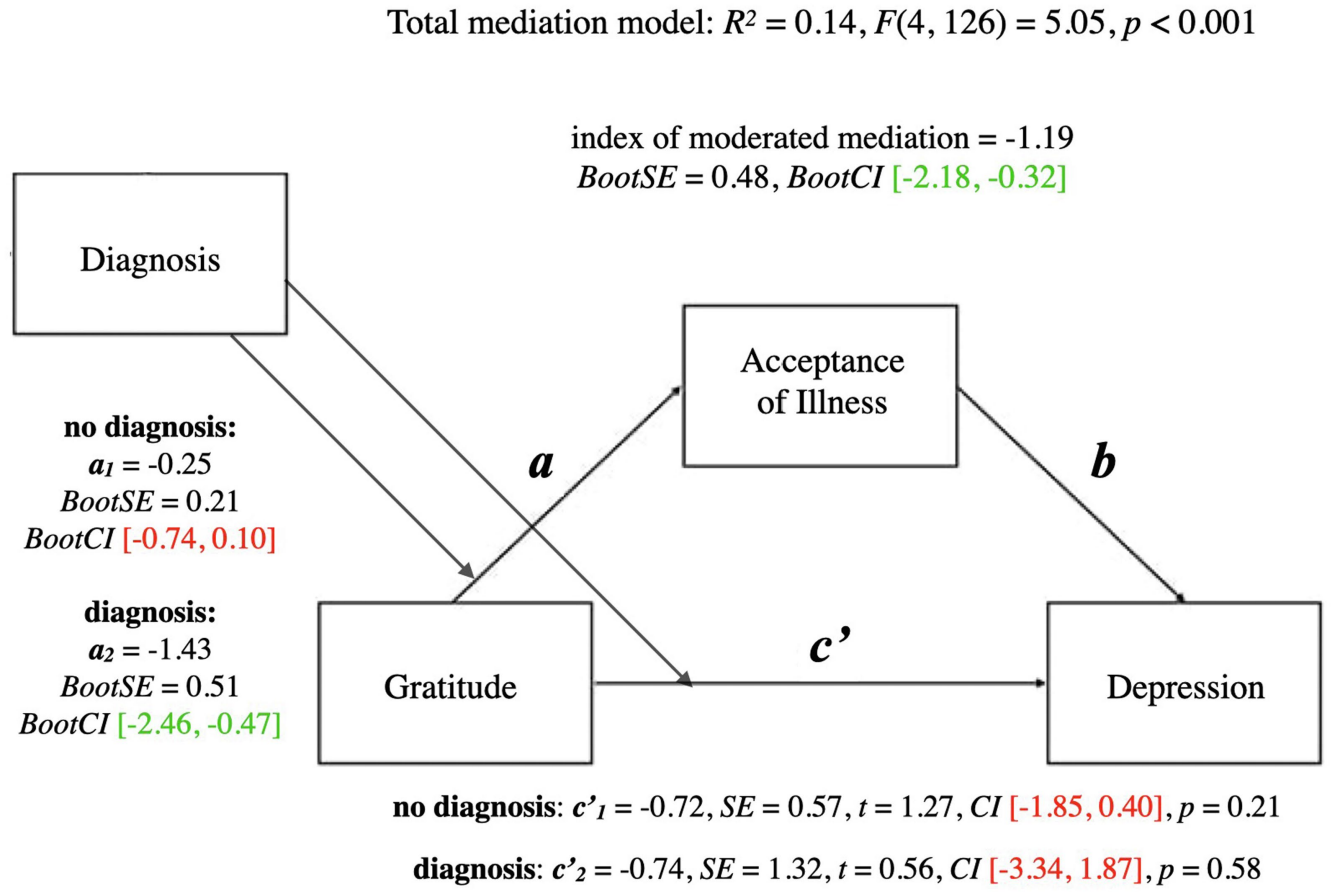
Moderated mediation model for gratitude and depression with diagnosis as moderator. a, conditional indirect effect of gratitude on wellbeing through acceptance of illness; c', conditional direct effect of gratitude on wellbeing index of moderated mediation = difference between conditional indirect effects CI marked green indicate significance; red – lack of significance.

A summary of the moderated mediation model for depression is presented below. For coefficients and additional statistics, see Figure 6 and Table 5.

The total mediational model for depression was significant (*p* < 0.001). The conditional direct effect of gratitude on depression was not significant either for people who had not been diagnosed as depressed (*p* = 0.21), or people who had been diagnosed (*p* = 0.58).

Similar to the previous analyses, the conditional indirect effect of gratitude on depression through acceptance of illness was significant for people who had been diagnosed as depressed at some point in their lives, (*BootCI* [−2.46, −0.47]), whereas it was not significant for people who had not been diagnosed (*BootCI* [−0.74, 0.10]). The index of moderated mediation was significant (*BootCI* [−2.18, −0.32]), indicating that the mediational relationships differed between the two groups. Similar to the results of the analyses of wellbeing and anxiety, acceptance of illness mediated the relationship between gratitude and depression for people who had received a formal diagnosis of depression, whereas acceptance of illness did not mediate the relationship between gratitude and depression for people who had not ever received a formal diagnosis of depression.

## Discussion

Our findings were consistent with our expectations. As expected, we found that dispositional gratitude was positively related to mental health, something that is consistent with research demonstrating that dispositional gratitude can serve as a protective factor for mental health (Ruini, 2017; Cregg and Cheavens, 2021). Also as expected, we found that acceptance of illness mediated relationships between gratitude and wellbeing, gratitude and depression, and gratitude and anxiety. Finally, we found that these relationships were moderated by whether someone had ever received a formal diagnosis of depression. We address these three findings below.

### At-Risk for Depression as a Context for the Present Results

To provide a more informed context for considering the present results, we briefly discuss the criteria we used to select our sample. As noted previously, we selected participants based on their CES-D scores, limiting participants to those who scored 20 or above. Such a cutpoint is often referred to as a “caseness cutpoint.” Scores above a caseness cutpoint indicate an increased risk for experiencing clinical depression, whereas variability in scores below the caseness cutpoint is unrelated to the likelihood that a person will experience an episode of clinical depression. Although there has been some debate over the years about exactly what the caseness cutpoint should be, there is little debate that 20 can be used as a caseness cutpoint (Vilagut et al., 2016).

A score above the caseness cutpoint does not in and of itself indicates that a person is clinically depressed. This needs to be confirmed by a formal diagnosis. Regardless, scores above the caseness cutpoint can be interpreted as indicators of distress. Moreover, the more people are above the cutpoint, the greater their distress and risk for an episode of clinical depression, whereas the extent to which people are below the caseness cutpoint is unrelated to their distress. Such a model is often referred to as a “discontinuity model.” Consistent with this reasoning, Nezlek et al. (1994) found that CES-D scores were negatively related to the quality and quantity of daily social interactions for individuals who were above the caseness cutpoint, whereas CES-D scores were unrelated to the quality and quantity of daily social interactions for individuals who were below the caseness cutpoint.

### Gratitude and Mental Health

The zero-order relationships (correlations) we found between gratitude and our measures of mental health are consistent with the large and growing body of research that has found positive relationships between gratitude and various measures of mental health (Wood et al., 2010; Allen, 2018; Portocarrero et al., 2020). Nonetheless, to our knowledge, such relationships have not been found among women who are at-risk for depression, or for that matter, among men who are at-risk for depression.

The zero-order relationships we found are important because they expand the types of distress on which gratitude may have an effect. Moreover, previous research has not examined relationships between gratitude and mental health separately for individuals who were and were not at-risk for depression. Assuming that some individuals in previous studies were sufficiently distressed so they would be classified as at-risk for depression, it is possible that the positive relationships found in these studies were due primarily to relationships among individuals who were not at-risk, i.e., the effects of gratitude on mental health do not occur when people are severely distressed. The present results clearly indicate this is not the case.

### Gratitude and Acceptance of Illness

The zero-order relationship (positive correlation) we found between gratitude and acceptance of illness also merits discussion. To our knowledge, previous research has not examined such relationships, and the present results expand the nomological network of gratitude. Our results suggest that being grateful makes it easier for people to accept the realities of distressing conditions such as illness or depression. Gratitude entails appreciating what a person has in life, and it appears that the more a person is able to do this, the more she can view her distress adaptively. This might include more recognition by someone that although she is suffering, she is not defined by her illness. Yes, illnesses create problems and distress, but if these problems and distress are viewed within a context that includes an appreciation of the positive aspects of a persons’ life, they may be seen as less problematic or distressing than if they are not seen this way.

### Acceptance of Illness and Mental Health

We also found zero-order relationships between acceptance of illness and mental health, including wellbeing, depression, and anxiety. Acceptance of illness encompasses the understanding that one’s circumstances are unfavorable and adjusting to the limitations that they impose. It is associated with high levels of self-awareness, insight into one’s situation, and a readiness to learn new ways of coping. Although defense mechanisms such as denial may interfere with treatment and delay recovery, acceptance of illness may have exactly the opposite effect. Our findings suggest that accepting one’s condition is an important part of recovery, as it promotes better psychological functioning and protects wellbeing.

### Acceptance of Illness as a Mediator of Relationships Between Gratitude and Mental Health

Although relationships between gratitude and mental health have been studied for almost 20 years, how gratitude leads to improved mental health is not well understood (Cregg and Cheavens, 2021). We found that acceptance of illness mediated relationships between gratitude and mental health, a finding that we believe can help to understand how gratitude promotes mental health and wellbeing. Previous research has found that feeling grateful may promote the use of adaptive coping strategies, which allow people to deal with difficult situations more effectively and to return to better levels of functioning more quickly (Emmons and Mishra, 2011). The possibility that adaptive coping may mediate relationships between gratitude and wellbeing was demonstrated by Tomczyk et al. (2021). In a sample of women with breast cancer, they found that task- and socially oriented coping mediated relationships between gratitude and mental health.

Within this context, the present results suggest that acceptance of illness may be a type of adaptive coping mechanism. People who are suffering accept the fact that they have a condition/illness, and they try to move on with their lives despite this. It appears that gratitude facilitates such beliefs and that in turn, such beliefs lead to enhanced mental health. We should note that Tomczyk et al. (2021) did not find what is considered to be the use of maladaptive coping strategies such as distraction mediated relationships between gratitude and mental health.

### Diagnostic History as a Moderator of How Acceptance of Illness Mediates Relationships Between Gratitude and Mental Health

On an exploratory basis, we examined how the mediational relationships we found might vary as a function of a participant’s diagnostic history. Had the participant ever been diagnosed as depressed? We examined this because individuals who have been diagnosed as depressed have a concrete, tangible explanation for or about their distress. A diagnosis provides people with a context for understanding their distress. Even if they are not currently receiving treatment (none of our participants was receiving treatment), people who have been diagnosed with depression at some point know what their feelings of distress represent, and they know what to expect.

People who have never been diagnosed as depressed may wonder what is wrong with them. They may feel sad, hopeless, and so forth, but what does this represent or mean? They may not have clear expectations about the future. How long will this last, what other effects might it have, and so forth? Moreover, the questions on the AIS refer to illness, and participants who were not familiar with clinical depression as a condition/illness (i.e., participants who had never been diagnosed) may have not been able to answer the questions in terms of their current level of distress. They do not have a basis to think of themselves as ill.

Given the uniqueness and novelty of this moderated mediation, we prefer not to speculate about the mechanisms that may be responsible for this effect. The effect was consistent across our three measures of mental health, providing some basis for generalizability; however, the present results are only preliminary.

### Theoretical Implications of the Study

We believe that our findings may be helpful in understanding how women at-risk of depression function. A popular model of depression states that depression is a “disorder of impaired emotion regulation” (Joormann and Gotlib, 2010, 1). People differ in what kinds of emotion regulation strategies they habitually choose to cope with negative events. Those who often choose maladaptive strategies, such as rumination, are at great risk of developing depression (D’Avanzato et al., 2013). A grateful disposition may be the opposite quality to a ruminative tendency, as it encourages a person to look for and dwell on the positive aspects of their lives. The conclusions from our mediation analyses suggest that this kind of disposition is related to higher acceptance of one’s situation, which in turn is related to decreased depressive symptoms. In the light of the emotion regulation concept, just as rumination predicts the onset of depression (Nolen-Hoeksema et al., 2008), gratitude may be associated with reduced vulnerability to depression through increased acceptance. The at-risk population that we studied was inherently vulnerable to developing major depressive disorder due to their elevated symptoms—which is when the strategies of emotion regulation may have a decisive role to play. We found that gratitude was related to wellbeing in this population, and there is research that suggests that the higher the wellbeing, the more effective the use of adaptive emotion regulation strategies such as reappraisal (McRae et al., 2012). Therefore, gratitude as a disposition may not only be one of those individual differences that protect mental health, but also it may help build the right environment for the application of adaptive emotion regulation strategies.

Some research suggests that one of the factors of depression vulnerability is people’s tendency for cognitive distortions and focusing attention on negative stimuli (Koster et al., 2017). Gratitude naturally directs attention to positive stimuli, which may help break the vicious cycle of automatic negative thoughts leading to depression (Beck, 1979). The potential role that gratitude may play in preventing depression should not be underestimated and we believe that our findings help extend the understanding of this role. We discuss the practical implications of this study in the subsection below.

### Practical Implications of the Study

At the foundations of positive psychology lies the principle that the aim of psychology should not only be to alleviate distress, but also to build skills and resources that nurture an individual’s wellbeing (Seligman, 2002). Our findings suggest that gratitude is a trait that may promote both outcomes at the same time. This study explored possible mechanisms underlying these relationships in an attempt to explain how gratitude works for the benefit of women at-risk of depression. The finding that gratitude in women with symptoms of depression is related to an attitude of acceptance toward their circumstances and that this may promote mental health outcomes has practical implications for depression treatment.

First of all, the study provides the rationale for further development of a gratitude intervention that would be specifically targeted at women at-risk of depression. Although our results do not demonstrate causation, they are consistent with recent research that suggests that gratitude can lead to the improved wellbeing of women who are experiencing psychological distress. For example, gratitude has been recently found to increase wellbeing and decrease depression in women with breast cancer, along with promoting post-traumatic growth (Sztachańska et al., 2019; Tomczyk et al., 2021). Gratitude also has a role in developing post-traumatic growth in female victims of poverty and abuse (Mushonga et al., 2021). Dispositional gratitude was negatively related to changes in symptoms of depression in a population of female nurses over the course of 4 months (Hao et al., 2022).

As was discussed in the Introduction, depression in women differs in its etiology, presentation of symptoms, and factors that contribute to the deterioration of mental health (e.g., Sloan and Sandt, 2006; Albert, 2015). There exist gratitude interventions that are effective in samples with clinical depression (Sin and Lyubomirsky, 2009), but it would be interesting to see one tailored to the needs of this specific population. Gratitude interventions are cost-effective, widely available, and easy to self-administer; therefore, they might be a good choice for women who are at-risk of developing clinical depression—as a self-help tool of choice before starting professional therapy or psychiatric treatment.

Second, one of the goals of therapy is to help patients develop skills and resources that will enable them to live and function with their condition. The findings of our study suggest that gratitude may constitute such a resource. People who suffer from depression usually experience automatic negative thoughts (Beck, 1979)—in therapy, they learn to cognitively restructure them (Ackerman, 2021), which promotes adjustment. It is worth noting how gratitude diary and similar positive practices are often used by cognitive-behavioral therapists as between-session homework (Davis et al., 2016). This is because gratitude naturally aids the therapeutic process of cognitive restructuring, or positive reappraisal (Lambert et al., 2012). This explanation for this mechanism of gratitude is in line with the conclusions drawn from our study. If gratitude is related to people’s attitude of acceptance of their circumstances, which protects mental health—we recommend future research of these relationships, which may 1 day lead to incorporating gratitude into mental health care programs.

### Limitations and Future Directions

Although we believe that this study contributes to our understanding of how gratitude relates to the mental health of women with depressive symptoms, it has certain limitations that can be addressed in future research. First, we used acceptance of illness as the mediator in our models. It is worth considering other mediators that could play an important role in explaining the mechanism of gratitude. To the best of our knowledge, it is the first study that examines any adjustment-related mediator of the relationship between gratitude and wellbeing in a population with depressive symptoms. In healthy populations, possible mediators of this relationship that were previously studied are, for example, self-esteem (Lin, 2015; Yildirim et al., 2019), spirituality (Bali et al., 2022), social support, and resilience (Kong et al., 2021). We recommend exploring these relationships in a population with depression, especially that, for example, social support is known to be a protective factor in developing depression (Gariépy et al., 2016).

Second, the sample chosen for this study consisted solely of women. Our intent was to study depression among women as a specific phenomenon. It would be interesting to replicate this study in a sample of men who were at-risk for depression, to see if gratitude works in the same way in both sexes. In a study by Yue et al. (2017), gratitude mediated the relationship between sex and depression, to women’s advantage. Even though depression is twice as prevalent among women than it is among men, women’s greater dispositional gratitude could be a protective factor in the development of depression. We should note, however, that a recent meta-analysis found no gender differences in how gratitude affects wellbeing (Portocarrero et al., 2020).

Third, we would also recommend collecting a larger sample that would provide a basis for examining how the relationships we found might vary as a function of demographic factors such as employment status, marital status, and so forth. Our exclusion criteria were mainly related to mental health and treatment because that was most important from the perspective of our research question and study design. Considering more inclusion and exclusion criteria could ensure a more homogenous sample in terms of demographic factors, and therefore allow for more generalizability of the findings.

Estimating the power of mediation and moderated mediation designs is quite complicated and requires numerous assumptions about different parameters (Fritz and MacKinnon, 2007). As a proxy of power analysis for mediation and moderated mediation, we have calculated power for multiple regression with 3 predictors, and the actual power was 0.95. It suggests that we have enough statistical power to draw accurate conclusions about the studied population using the data we have collected. We also performed a power analysis for indirect effects in R (Schoemann et al., 2017) with Monte Carlo simulation (1,000 replications). For a model with one mediator, the results showed the power of 0.76 (*p* < 0.05) for our sample of 131.

Although our study is not underpowered, future researchers are encouraged to remember the concerns associated with small sample size. Small sample sizes may produce bigger effects than a larger sample would, due to the small sample bias (White et al., 2019). They also increase the chance of accepting false premises or generating false-positive results (Faber and Fonseca, 2014). Large samples ensure a bigger internal and external validity of the study (Faber and Fonseca, 2014); therefore, we recommend that future research takes it into consideration.

Moreover, we used self-report measures because we were interested in how people felt and how they subjectively evaluated their circumstances. Although self-report measures are widely used in psychological research as they allow us to gain insight into people’s emotional processes, there are concerns one needs to be aware of. They may pose a threat of social desirability or other response distortions (Paulhus, 1991), which may be the cost to pay for its indisputable advantage of anonymity, especially in a sensitive sample such as women at-risk of depression. We do encourage, however, the use of repeated measures such as ambulatory assessment or daily diaries to study the relationships that were the subject of this paper, as it could address some of the reliability and validity issues associated with single-measure self-report studies (Krejtz et al., 2016; Nezlek et al., 2017; Sztachańska et al., 2019; Nezlek, 2020).

We used CES-D to screen for depression, following the guidelines of Park and Yu (2021) who concluded their meta-analysis stating that “the CES-D, which has shown high diagnostic accuracy in adults, can be recommended for use as a first-stage screener for MDD” (Park and Yu, 2021, 1). We encourage researchers to further examine the relationships we found with another validated screening tool, such as, for example, Beck Depression Inventory II (Beck et al., 1996).

We also need to acknowledge that the reliability of the scale we used to measure wellbeing was lower than the other scales used in this study. Nevertheless, Shrout (1998), a widely cited guideline for evaluating reliability, suggests that Cronbach’s alphas from 0.61 to 0.80 indicate *moderate reliability*, so the reliability of wellbeing (0.66) falls within this range. By definition, unreliability is a form of error variance, and using less reliable measures makes it more difficult to find relationships than using more reliable measures. Therefore, we found relationships involving wellbeing despite its lower reliability. Given that we found all the relationships involving wellbeing that we expected to find, if the wellbeing measure had been more reliable, perhaps these relationships would have been stronger.

Finally, as far as future research directions are concerned, we encourage testing the relationships we have found in the light of the COVID-19 pandemic. The pandemic has led to a dramatic increase in the prevalence of depression (by almost 30%; Daly and Robinson, 2022). In this distressing time, studying the psychological resources that might promote mental health is extremely important (Robinson et al., 2022). For example, social support and optimism may be such resources (Chen and Bonanno, 2020). But most importantly, research in pandemic coping has provided evidence for the protective role of gratitude in a psychological crisis. A disposition of gratitude was related to higher resilience and a more adaptive reaction to negative events related to the pandemic, while it also predicted lower levels of depressive symptoms and perceived stress (Wolfe, 2021). Gratitude was also studied as a coping style that leads to favorable outcomes in the threatening time of the COVID-19 pandemic (Jans-Beken, 2021). Although our study did not consider the circumstances of the pandemic, it considered a population at a fragile stage of life with compromised wellbeing. As women’s mental health is more affected by the pandemic than men’s (Santomauro et al., 2021), we recommend looking into the potential role of gratitude in building adjustment and wellbeing of women at-risk of depression who struggle with new threats and challenges that the pandemic has posed. We believe that gratitude has the potential to improve the functioning of this population and more empirical research is needed to verify a causal link between gratitude and wellbeing.

## Conclusion

As depression is known to be the most common mental health problem in women (World Health Organization, 2021), we believe it is particularly important to understand what dispositional factors might be related to women’s better mental health. We found that dispositional gratitude is positively related to mental health (depression included) through the acceptance of illness. The finding that gratitude in women with symptoms of depression is related to an attitude of acceptance toward their circumstances and that this positively influences their mental health outcomes has practical implications for depression treatment. We recommend that further research focus on designing gratitude interventions specifically tailored to the needs of women at-risk of depression, as gratitude might have the potential to protect and promote mental health in this population.

## Data Availability Statement

The original contributions presented in the study are publicly available. This data can be found here: https://osf.io/dvef3/?view_only=0833c094d6eb472e876cfa1d9b76c512.

## Ethics Statement

The studies involving human participants were reviewed and approved by The Ethics Committee of SWPS University of Social Sciences and Humanities, Warsaw, Poland. The patients/participants provided their written informed consent to participate in this study.

## Author Contributions

JT designed and conducted the study and drafted the manuscript. IK supervised the study and the procedure planning process. JBN provided conceptual and methodological guidance. JT and JBN performed the data analyses and IK helped with data interpretation. JBN and IK edited and reviewed the manuscript. All authors approved the version of the manuscript to be published and agreed to be accountable for all aspects of the work.

## Funding

This research was funded by the National Science Center, Preludium grant 2016/21/N/HS6/02840 to JT. Open access of this article was financed by the Ministry of Science and Higher Education in Poland under the 2019-2022 program “Regional Initiative of Excellence,” project number 012/RID/2018/19.

## Conflict of Interest

The authors declare that the research was conducted in the absence of any commercial or financial relationships that could be construed as a potential conflict of interest.

## Publisher’s Note

All claims expressed in this article are solely those of the authors and do not necessarily represent those of their affiliated organizations, or those of the publisher, the editors and the reviewers. Any product that may be evaluated in this article, or claim that may be made by its manufacturer, is not guaranteed or endorsed by the publisher.
